# Ectopic beats arise from micro-reentries near infarct regions in simulations of a patient-specific heart model

**DOI:** 10.1038/s41598-018-34304-y

**Published:** 2018-11-06

**Authors:** Rafael Sachetto Oliveira, Sergio Alonso, Fernando Otaviano Campos, Bernardo Martins Rocha, João Filipe Fernandes, Titus Kuehne, Rodrigo Weber dos Santos

**Affiliations:** 1 Department of Computer Science, Universidade Federal de São João del-Rei, São João del-Rei, Brazil; 2 Graduate Program in Computational Modeling, Universidade Federal de Juiz de Fora, Juiz de Fora, Brazil; 3 Department of Physics, Universitat Politècnica de Catalunya, Barcelona, Spain; 4 Institute for Computational and Imaging Science in Cardiovascular Medicine, Charité – Universitätsmedizin, Berlin, Germany; 5 Department of Congenital Heart Disease, German Heart Centre Berlin (DHZB), Berlin, Germany; 6 School of Biomedical Engineering and Imaging Sciences, King’s College London, London, UK

## Abstract

Ectopic beats are known to be involved in the initiation of a variety of cardiac arrhythmias. Although their location may vary, ectopic excitations have been found to originate from infarct areas, regions of micro-fibrosis and other heterogeneous tissues. However, the underlying mechanisms that link ectopic foci to heterogeneous tissues have yet to be fully understood. In this work, we investigate the mechanism of micro-reentry that leads to the generation of ectopic beats near infarct areas using a patient-specific heart model. The patient-specific geometrical model of the heart, including scar and peri-infarct zones, is obtained through magnetic resonance imaging (MRI). The infarct region is composed of ischemic myocytes and non-conducting cells (fibrosis, for instance). Electrophysiology is captured using an established cardiac myocyte model of the human ventricle modified to describe ischemia. The simulation results clearly reveal that ectopic beats emerge from micro-reentries that are sustained by the heterogeneous structure of the infarct regions. Because microscopic information about the heterogeneous structure of the infarct regions is not available, Monte-Carlo simulations are used to identify the probabilities of an infarct region to behave as an ectopic focus for different levels of ischemia and different percentages of non-conducting cells. From the proposed model, it is observed that ectopic beats are generated when a percentage of non-conducting cells is near a topological metric known as the percolation threshold. Although the mechanism for micro-reentries was proposed half a century ago to be a source of ectopic beats or premature ventricular contractions during myocardial infarction, the present study is the first to reproduce this mechanism in-silico using patient-specific data.

## Introduction

A myocardial infarction (MI) usually involves the occlusion of a coronary artery of the ventricle or other types of coronary artery diseases. In the acute phase, ischemia and necrosis in the injured region lead to the deterioration of electrical activity at the cellular and tissue levels^1,2^. Early revascularization and new medicaments greatly improve survival after MI. During the healing phase of MI, fibrosis occurs to replace necrotic cells^3^. However, post-MI patients remain at substantial risk for ventricular arrhythmia^4^. In fact, most deaths in post-MI patients are due to arrhythmias and recurrent MI, i.e., a second episode of an acute MI in the same region of the first^5^. Variant and unstable angina are also manifestations of different coronary artery diseases, such as a vasospasm versus a partial obstruction, which have recurrent episodes of chest pain at rest and the development of ventricular arrhythmias in common^6^. As a result of multiple episodes, injured tissue is highly heterogeneous where both fibrosis (one week old or older episodes) and necrosis (more recent episodes) co-exist^7^.

Although different in many aspects, the aforementioned myocardial infarctions have heterogeneous injured tissues that actively participate in both the generation and maintenance of cardiac arrhythmias in common. Experimental and clinical findings have suggested that the main trigger of a variety of these arrhythmias is focal excitations, which can lead to premature ventricular complexes, or ectopic beats, that are co-located within the infarct and ischemic regions of the heart^8–10^. In addition, as mentioned before, the injured tissue has an important feature that is exploited in this work: the presence of ischemic myocytes next to non-conducting cells (fibrosis in the case of recurrent MI, necrosis in the case of a first acute MI, or both).

Today, there are three main hypotheses for the mechanisms of ectopic beat formation: abnormal automaticity, triggered activity and micro-reentries^11–13^. However, until now, none of these mechanisms have been fully understood nor elucidated. In this work, we combine computational models and non-invasive imaging techniques of the heart to study the mechanism of micro-reentries. This mechanism was first described and proposed half a century ago as a result of clinical and animal studies^13–15^. In particular, Boineau and Cox^13^ presented a detailed hypothesis based on experimental data that linked electrophysiology findings, such as fractionated electrograms, to the heterogeneity of acute infarct tissues. The micro-reentry mechanism may be related to the zig-zag course of activation that occurs due to the intricate morphology of a two-phase medium composed of conducting (myocytes) and non-conducting (necrosis or fibrosis) materials. This two-phase medium forms a maze in which waves fractionate and follow zig-zag pathways. Depending on the topology of this maze, intricate circuits can be formed that permit sustained activation, via re-entries, inside the infarct region. In addition, this electrical activity trapped inside the infarct region may continuously or transiently escape and generate ectopic beats. This escape occurs whenever the activity crosses the peri-infarct zone (PZ), i.e., the interface between the injured region and surrounding healthy cardiac tissue.

Unfortunately, a final demonstration that re-entry is the underlying mechanism by which an infarct or ischemic region becomes an abnormal source of fast-pacing stimuli, i.e., an ectopic pacemaker, is still pending. While fractionated electrograms have been frequently observed near the infarct region, combined and simultaneous characterization of the underlying heterogeneous 3D structure poses a great challenge for experimental studies. Biological variability defies reproducibility, and the micro-scale of the cardiac structure cannot currently be determined using *in-vivo* microscopy tools.

In this computational study, we combined different methods and models to simulate, for the first time, the generation of ectopic beats near infarct regions via the micro-reentry mechanism in a patient-specific biventricular model. The simulation results reveal that ectopic beats emerge from micro-reentries that are sustained by the heterogeneous structure of the infarct regions. The collection of results also suggests conditions for the generation of ectopic beats in terms of degree of tissue heterogeneity and level of ischemia. Therefore, we believe the results presented in this work are an important step toward the validation of the hypothesis that micro-reentries may be the mechanism by which an infarct or ischemic region becomes an abnormal source of ectopic beats.

Similar to previous works, the uncertainty related to the topological structure of the heterogeneous tissue during myocardial infarction was treated via Monte Carlo simulations^16–27^. In this work, as in previous computational studies, non-conducting material was also related to fibrosis^26–31^. The excessive deposition of collagen in the extracellular matrix of injured tissue may physically and electrically separate neighboring myocytes or completely replace dead myocytes^32,33^. Late gadolinium-enhanced Magnetic Resonance Imaging (LE-MRI) is a new and commonly used non-invasive exam used for detecting regions of fibrosis, as well as the severity of its extension, in patients who have experienced a myocardial infarction^34^.

The key methods and models are: 1) an accurate model of the anatomy of the heart, as well as the location and geometry of both the infarct and PZ regions, which were generated by processing images of the late-enhancement MRI of a patient who has experienced a recent myocardial infarction; 2) modern mathematical models of the electrophysiology for healthy and ischemic cells; 3) Monte Carlo simulations to identify which levels of non-conducting cells and ischemia of the infarct region have a high probability to generate micro-reentries, and, in particular, the use of previous theoretical results that related fractions of non-conducting cells, reentries and the percolation threshold to allow us to significantly reduce the number of simulations^26–31^; and 4) a modern and efficient parallel cardiac simulator that ran hundreds of complex 3D simulations within a feasible amount of time^35^.

## Methods

### Generation of a patient-specific heart model based on cardiac MR imaging

#### Patient Data

Data from a single patient were used to build the computer models in this proof of concept study. The main criteria used for patient selection were to be diagnosed with a recent myocardial infarction and to have cardiac images that could be used to represent the heart geometry as well as the infarct location, size and geometry. The chosen patient used in this work fulfilled these main requirements. In addition, this patient also had two different infarct regions, with distinct locations, sizes and geometries. This enabled us to study how the size of the infarct region influences the whole phenomenon.

Coronary angiography was performed in the patient (58 years old; weight, 80 kg; height, 170 cm) two months before the cardiac MRI study and revealed a 50% stenosis of the proximal RCX. An intervention was not performed. ECG at rest showed a sinus rhythm, PQ 136 ms, QRS 100 ms, QT/QTc 370/445 ms and descending ST and pre-terminal negative T wave in II, III and avF. The patient also had moderate aortic valve stenosis (gradient by Doppler, 50 mmHg). Cardiac MR imaging was performed on the whole body using a 1.5 Tesla scanner (Achieva R 3.2.2.0, Philips Medical Systems, Best, The Netherlands) with a five-element cardiac phased-array coil. The three-dimensional anatomy of the ventricles was determined using the balanced turbo field echo cine short axis and 3D whole heart scans. The contrast media, Gadolinium, was administered for late-enhancement and T1-mapping to visualize the scars and measure extracellular volumes (ECV), which correlated with myocardial fibrosis. Left ventricle function and tissue structure was characterized by MRI: EF 40%, LV EDV 88 ml/m^2^ and ECV = 26%. In this study we used retrospective anonymized data. The Clinical Ethics Committee at the DHZB (German Heart Center Berlin), following the ethical guidelines of the 1975 Declaration of Helsinki, approved the study, under the name of Cardioproof, and granted the use of retrospective data without informed consent of patients for anonymized data.

#### Image Segmentation

**Figure 1:**
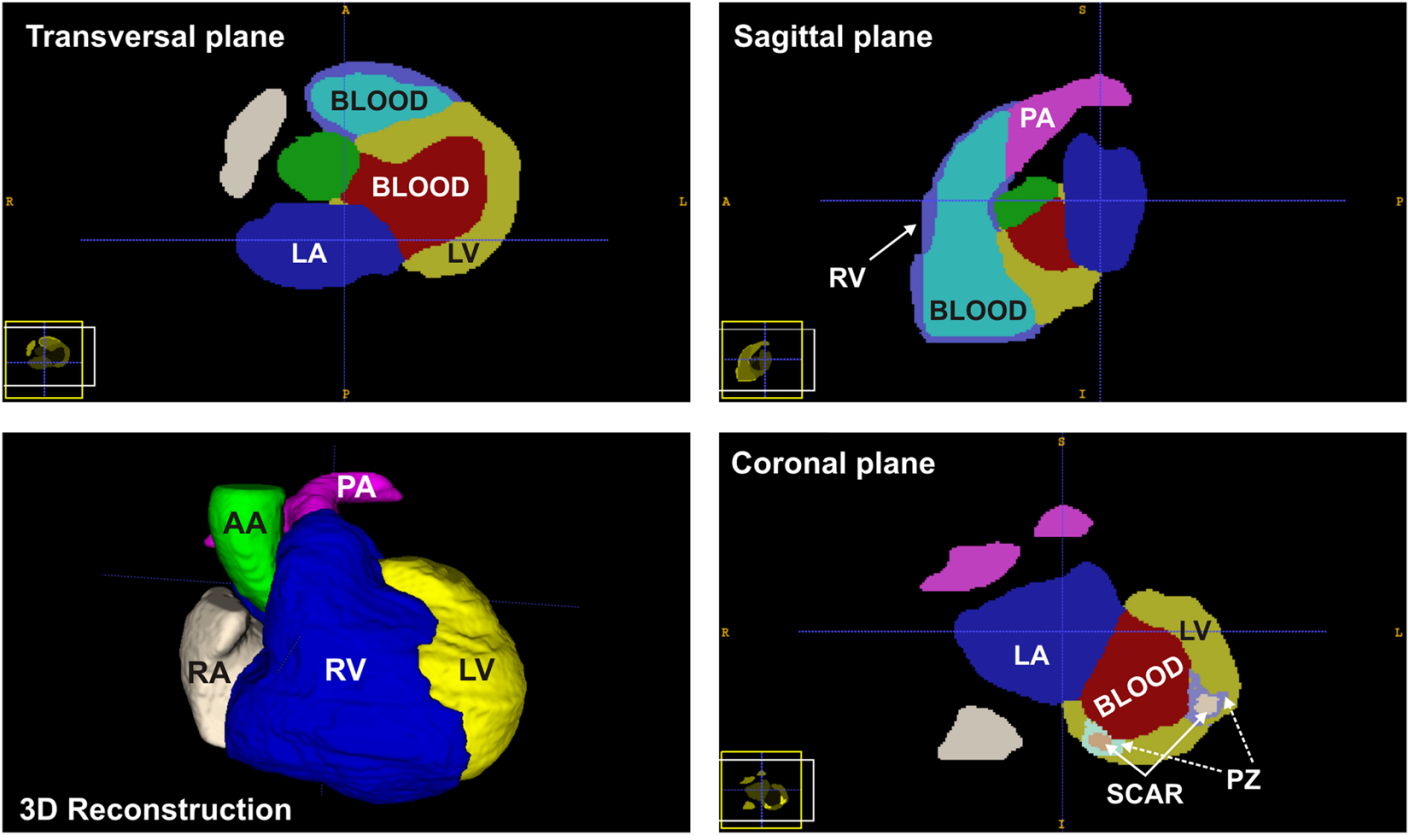
Segmented MR image. Representative 2D slices in the three main planes and 3D reconstruction are shown. Labels: right atrium (RA), left atrium (LA), right ventricle (RV), left ventricle (LV), aorta artery (AA), pulmonary artery (PA) and peri-infarct zone (PZ).

An 8-label structural segmentation of the ventricles (left and right), blood pools, atria (left and right), aortic root and pulmonary artery was performed via a semi-automatic, watershed 3D-based algorithm embedded in ZIBAmira (Zuse Institute Berlin, Germany). Late gadolinium-enhanced MR was used to image the myocardial scar. Infarct regions were defined based on their higher gray value intensity compared to the healthy myocardium. Next, the infarct region was subdivided into scar tissue and a PZ by creating a threshold for the images based on the intensity values of the voxels. We have considered as infarct regions those with signal intensity (SI) values 20% higher than the mean myocardial intensity. Within each infarct region, we labeled as PZ the regions with SI values lower than 50% of the maximum SI found in the corresponding infarct region. Because both structural and scar segmentations were based on images acquired during the same procedure, the MR sequences only had a slight misregistration, which was manually corrected. Thus, the new tags (scar and PZ zone) were directly incorporated into the structural segmentation, as presented in Fig. 1.

After the completion of the segmentation process for each region of interest, such as the left ventricle, scar and PZ zone, we extracted the surface mesh with the aid of the Seg3D filter isosurfaces. Then, with the surfaces defined in an appropriate format (STL), we embedded them in a bounding box region and applied an algorithm that considered the desired mesh resolution as the input. With this algorithm, we generated meshes to simulate cardiac electrical activity using the finite volume method. The different surfaces of the LV, PZ and SCAR were used to tag different volumes in their respective regions of the mesh. These tags identified the type of model used: LV referred to myocardium without lesions and with a normal electrophysiology; SCAR identified regions with severe perfusion deficits in a future, recurrent MI or upon the first acute MI episode. Therefore, the model for this region included myocytes with severe ischemia and a certain amount, *ϕ*, of non-conducting tissue (representing fibrosis in the case of a recurrent MI or necrosis for the first acute MI); PZ identified the ischemic border zone, a region with a moderate perfusion deficit in the case of a recurrent MI or the first acute MI^36,37^. Therefore, we modeled myocytes with moderate ischemia and a lower fraction of non-conducting tissue, *ϕ*_*PZ*_ < *ϕ*. The next section presents the further details of these models.

### Electrophysiology modeling

#### Cardiac myocyte modeling under normal and ischemic conditions

To perform the computational experiments, we used a modified ten Tusscher model of human ventricle myocyte^38^ (TT3), which is a simplified version of the original model^39^. In this particular model, the transmembrane potential (*V*) and ionic currents (*I*_*ion*_) are related as follows:

1 $$\begin{array}{lll}-{C}_{m}\frac{\partial V}{\partial t} & = & {I}_{ion}={I}_{Na}+{I}_{K1}+{I}_{to}+{I}_{Kr}+{I}_{Ks}+{I}_{CaL}\\  &  & +\,{I}_{NaCa}+{I}_{NaK}+{I}_{pCa}+{I}_{pK}+{I}_{bCa}+{I}_{bNa}+{I}_{KATP},\end{array}$$

where *Cm* is the membrane capacitance, and each ion channel, exchanger or pump on the right side depends on the transmembrane potential and gating variables^38^. Under normal conditions, Eq. (1) produces an action potential (AP) with a duration of approximately 300 ms.

To include the effects of ischemia, we modified the TT3 model to reproduce hypoxia, hyperkalemia and acidosis as described before^40,41^. Briefly, hypoxia was modeled by the inclusion of a *K*^+^ current activated by Adenosine Triphosphate (ATP) called I_*K*(ATP)_ and by modifying the *P*_*Ca*(*L*)_ conductivity to also be dependent on ATP. The formulations for *I*_*KATP*_ and *P*_Ca(*L*)_ are:

2 $$\begin{array}{rcl}{I}_{KATP} & = & {g}_{{K}_{ATP}}(V-{E}_{k}),\,{g}_{{K}_{ATP}}={G}_{{K}_{ATP}}{P}_{ATP}{([K{]}_{o}/{[K]}_{o,normal})}^{n},\\ {G}_{{K}_{ATP}} & = & 195\times {10}^{-6}/Nichol{s}_{area}(nS/c{m}^{2}),\\ {P}_{ATP} & = & \frac{1}{1+{(\frac{{[ATP]}_{i}}{{k}_{0.5}})}^{H}},\end{array}$$

where *E*_*k*_ is the potassium reversal potential, *Nichols*_*area*_ = 5 × 10^3^, *n* = 0.24, *H* = 2, *k*_0.5_ = 0.250 *μM* according to^40^ and

3 $${P}_{CaL,ATP}=\frac{1}{1+{(\frac{{k}_{0.5}}{{[ATP]}_{i}})}^{H}},$$

with *H* = 2.6, *k*_0.5_ = 1.4 *mM*.

**Figure 2:**
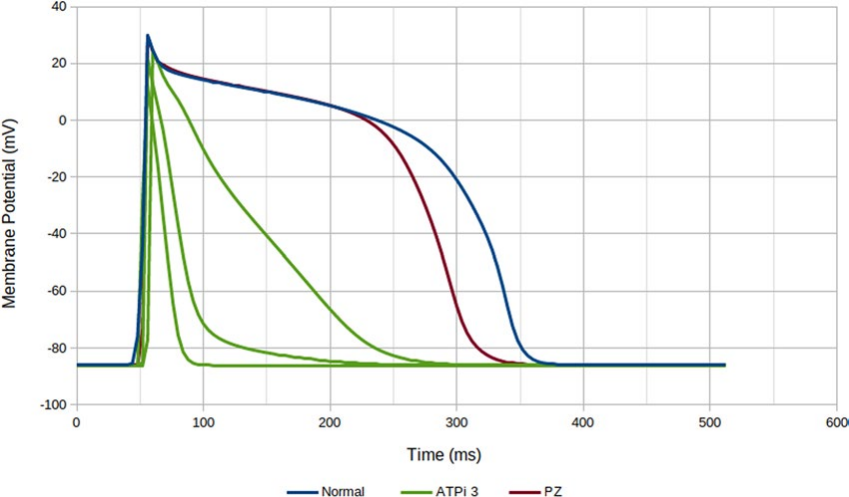
Dependence of the AP waveforms, obtained with the modified TT3 model, on the parameter [*ATP*_*i*_] to characterize the degree of hypoxia. AP waveforms were obtained at different points for the small wedge simulation (scar, PZ and healthy regions) with the pair (*ϕ* = 0, [*ATP*_*i*_] = 3 mM). In the same region with the same value of [*ATP*_*i*_], different waveforms were found due to the influence of the neighboring myocytes via electrotonic effects.

Hyperkalemia was introduced by increasing the extracellular potassium concentration [*K*_o_], and acidosis was modeled by reducing the L-type calcium and sodium current conductances (*g*_*cal*_ and *g*_*na*_, respectively). The behavior of our implemented model is in accordance with previous work regarding the conduction velocity (CV), resting membrane potential and APD changes^40–44^. Figure 2 presents how the level of [ATPi] (in a range of 3.0 to 6.8 mM) affects AP waveforms.

#### Modeling Action Potential propagation on heterogeneous cardiac tissue

The patterns of the two-phase media composed by non-conducting (fibrosis) and conducting cells inside infarct regions have been shown to be extremely complex^45^. Unfortunately, the non-invasive imaging techniques available today do not have the needed spatial resolution to reveal the underlying topology yet. Therefore, a solution is to artificially create many patterns via stochastic methods. In this work, we have performed this by randomly replacing conducting myocytes by non-conducting cells (fibrosis) for a given probability, *ϕ*. This same methodology was used before^16–18,21–27^ and has been usually referenced as diffuse fibrosis, characterized by the distribution of non-conducting tissue along normal myocytes and a lack of well defined compact blocks of non-conducting areas. Non-conducting and conducting tissues are interleaved and may form a complex maze.

We modeled diffuse fibrosis by randomly removing active tissue volumes of a regular size of (100 *μm*)^3^, and replacing it with non-conducting inert tissue. This block roughly represented a pack of 25 cardiac myocytes (each approximately 100 × 20 × 20 *μm*). This approach has been extensively employed to study reentries in cardiac tissue^16,17,21,22,25^. The maze of conducting tissue co-located within an inert fibrotic structure produced a fractionation of the AP propagation on active cardiac tissue, Ω_*A*_. This propagation was modeled with the monodomain model, a partial differential equation of the reaction-diffusion type:

4 $$\beta {C}_{m}\frac{\partial V}{\partial t}+\beta {I}_{ion}(V,\eta )=\nabla \cdot (\sigma \nabla V)+{I}_{stim},$$

5 $$\frac{\partial \eta }{\partial t}={f}(V,{\eta }),$$

where *V* is the transmembrane potential; *I*_*ion*_ is the total ionic current that may also depend on gating variables η, as described in Eq. (1); *β* is the surface-volume ratio; *C*_*m*_ is the membrane capacitance; *I*_*stim*_ is the current due to an external stimulus; and ***σ*** is the monodomain conductivity tensor. The model is further equipped with appropriate initial conditions and no–flux boundary conditions: σ∇*V*. **n** = 0 on ∂Ω_*A*_, where **n** is the normal vector of the surface, ∂Ω_*A*_. In this way, there is no exchange of currents between the active myocytes and non-conducting regions because, by definition, non-conducting regions do not belong to Ω_*A*_.

#### Numerical Methods

To solve the monodomain equations and simulate the action potential propagation in heterogeneous tissue, we used an efficient parallel cardiac solver^35^. This particular solver can handle non-uniform and non-conforming adaptive meshes. We used this feature to create a fine mesh (hexahedral segments of 100 *μm*) only in the damaged region (scar and PZ). For the rest of the mesh, discretization varied between 200 and 800 *μm*. The finite volume method was used to solve the partial differential equation (PDE), see Eq. (4), whereas the Rush-Larsen method was used to solve the ordinary differential equations (ODE) associated with the TT3 model. Both methods used a time discretization of 0.02 ms.

### Micro-reentry mechanism

**Figure 3:**
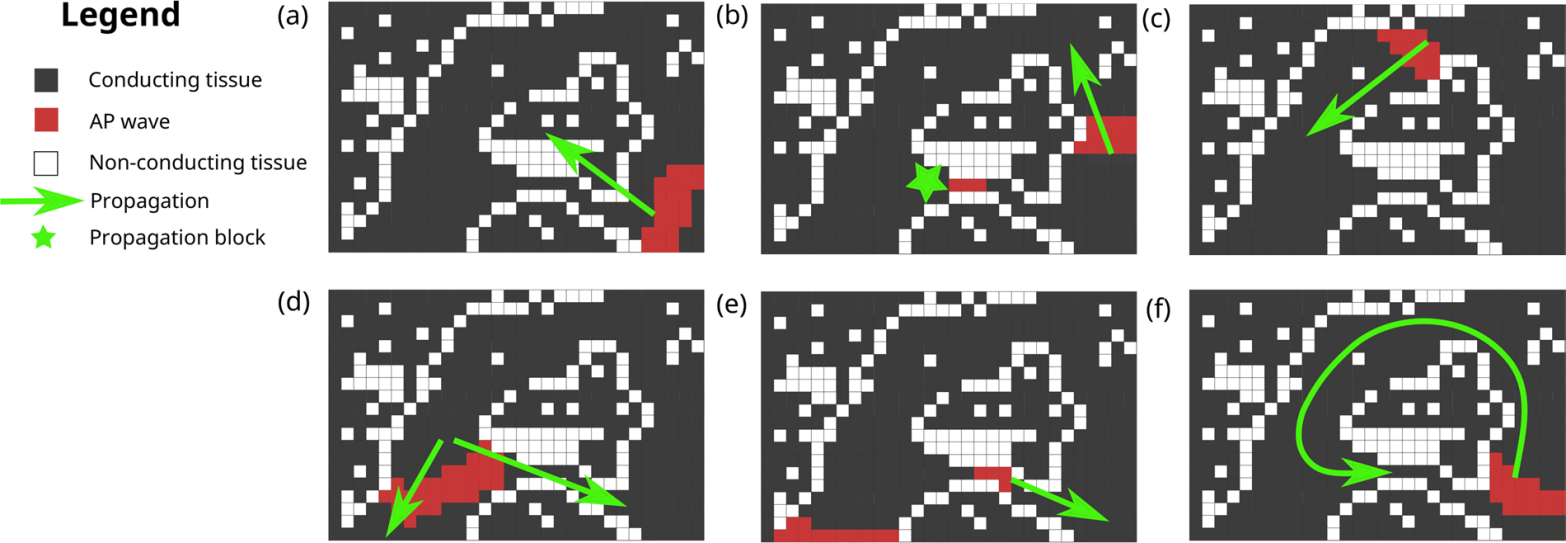
A schematic representation of the micro-reentry mechanism. The first necessary condition is the creation of a reentry path via a unidirectional block. Panel (b) presents this unidirectional block due to a source-sink mismatch. When the wave is confined in a thin tunnel (source), it does not have enough power to propagate the AP to the neighboring cells on the left when the thin tunnel suddenly opens (sink). The second necessary condition is presented in panel (e). When the wave reenters the thin path where it was blocked, it can only propagate if the cells are already repolarized, i.e., the wavelength has to be shorter than the created reentrant pathway.

In previous works, we associated the genesis of ectopic beats in 2D simulations of ventricular tissue and in 3D simulations of atrial tissue with the mechanism of micro-reentries^26,27^. Common features of these two studies revealed the minimum conditions for the creation of micro-reentries in heterogeneous tissues: 1) long reentrant paths within the damaged region; 2) short action potential and wavelength. Figure 3 illustrates the basic mechanism. The first condition for the creation of a reentrant path is a unidirectional block. As illustrated in Fig. 3(b), the unidirectional block is the result of a source-sink mismatch. The second condition is to have a wavelength shorter than the created reentrant pathway. Figure 3(e) shows that when the wave reenters the path where it was first blocked, the AP can only propagate if the cells are already repolarized.

In this work, diffuse fibrosis was used to generate patterns that could induce source-sink mismatches, unidirectional blocks and reentrant pathways. Other patterns of fibrosis exist, such as patchy, interstitial and compact^45^. Different patterns of fibrosis were modeled before and were able to generate long reentrant pathways^18,20,46^. In this work, we chose the simplest model, i.e., diffuse fibrosis, since this is enough to fulfill the first condition for the generation of micro-reentries: the creation of long reentrant pathways.

**Figure 4:**
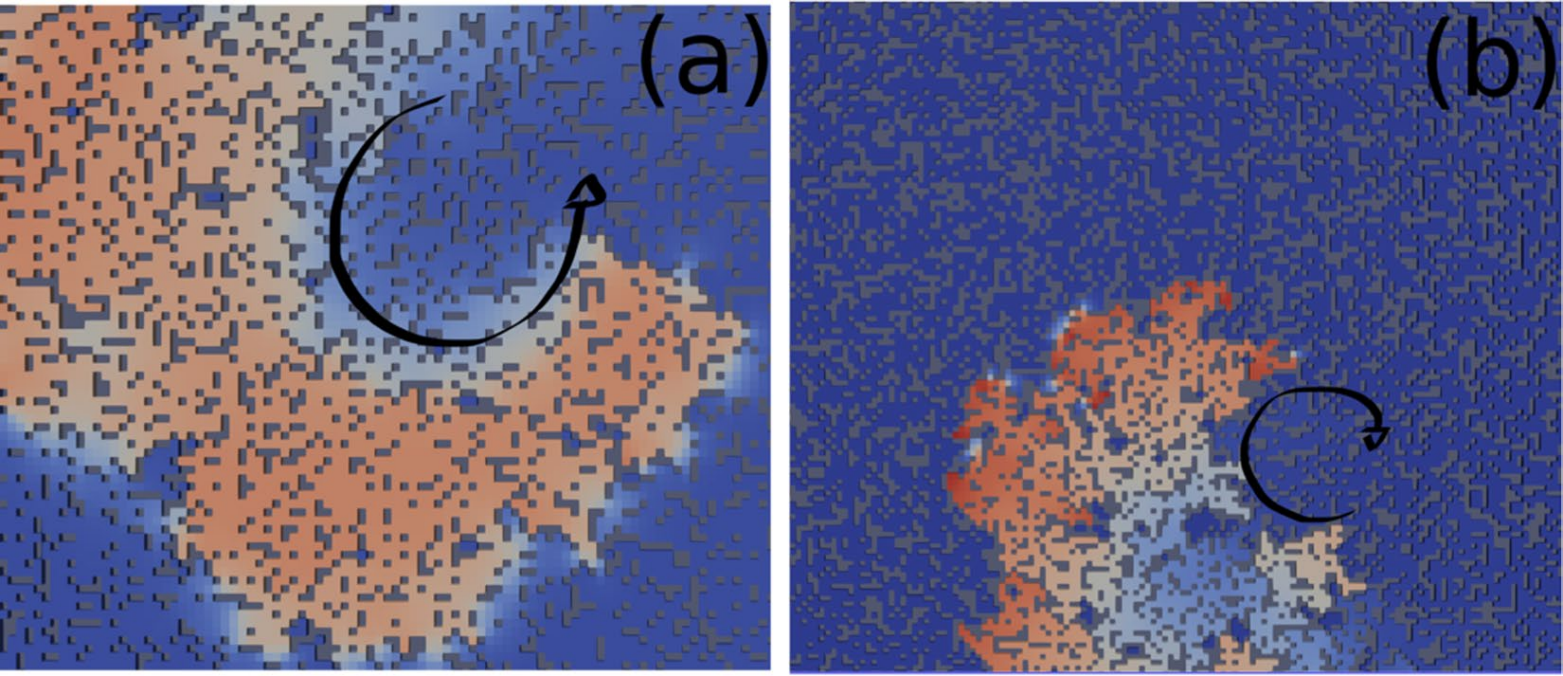
Two simulations on a thin slab of tissue with diffuse fibrosis. In both simulations, we observed sustained micro-reentries and that the wavelengths of the fractionated waves were similar. In Panel (a) we simulated ischemia with [*ATP*_*i*_] = 4 mM, [*Ko*] = 7 mmol/l and a 50% reduction of *g*_*CaL*_ and *g*_*Na*_, whereas in the simulation depicted in Panel (b), we only changed [*ATP*_*i*_] to 3 mM.

The second important ingredient is to have a wavelength (APD × CV) that is short enough to allow the wave to re-excite the location where the unidirectional block occurred. As previously described, ischemia decreases the wavelength by reducing both APD and CV. In fact, any of the described mechanisms that take place during ischemia, hypoxia, hyperkalemia or acidosis can reduce the AP wavelength alone. Therefore, many different combinations of hypoxia, hyperkalemia and acidosis can result in the same modified wavelength and, as a consequence, satisfy the second condition for micro-reentry. As an example, Fig. 4 presents the results of two simulations on a thin slab of tissue with diffuse fibrosis. In both simulations we observed sustained micro-reentries (see Supplementary Video 1) and that the wavelengths of the fractionated waves were similar. However, for the first simulation, as shown in Fig. 4(a), we had [*ATP*_*i*_] = 4 mM, [*K*_o_] = 7 mmol/l and a 50% reduction of *g*_*CaL*_ and *g*_*Na*_, whereas, for the second simulation, Fig. 4(b), we had only changed [*ATP*_*i*_] to 3 mM (hypoxia only). With these parameters, in the first simulation, we had APD = 100 ms and CV reduced to half of its normal value, whereas for the second simulation, we had APD = 50 ms and a normal CV, i.e., both simulations had the same wavelength.

Figure 4 suggests that similar results, in terms of propagating wavelength and induction of micro-reentries in heterogeneous media, were obtained with two different models: one that considered the three processes (hyperkalemia, acidosis and hypoxia) and the other that only considered hypoxia. Indeed, among hypoxia, hyperkalemia and acidosis, it has been shown before that hypoxia is the process that has the greatest influence on APD and wavelength during ischemia^40,41^. Therefore, for the next simulations, we chose the simplest model since it fulfills the second condition for the generation of micro-reentries, i.e., it can create short wavelengths, and it also reproduces AP waveforms with APD values that are usually found during acute ischemia^40^.

### Monte Carlo simulations

Even if a fast, state-of-the-art cardiac simulator^35^ is available, full heart or bi-ventricular 3D simulations could still be a time-consuming task for Monte Carlo studies. For this reason, we restrict our parameter space to the pair (*ϕ*, [*ATP*_*i*_]). According to the previous sections, two conditions were necessary for the mechanism of micro-reentries: long reentrant pathways and short wavelengths. The parameter *ϕ* was used to parameterize the non-conducting patterns. To reduce the amount of simulations, we parameterized the wavelengths during ischemia by [*ATP*_*i*_], since Fig. 4 shows that this choice has generated similar results in terms of propagating wavelength and induction of micro-reentries in an ischemic and heterogeneous media.

**Figure 5:**
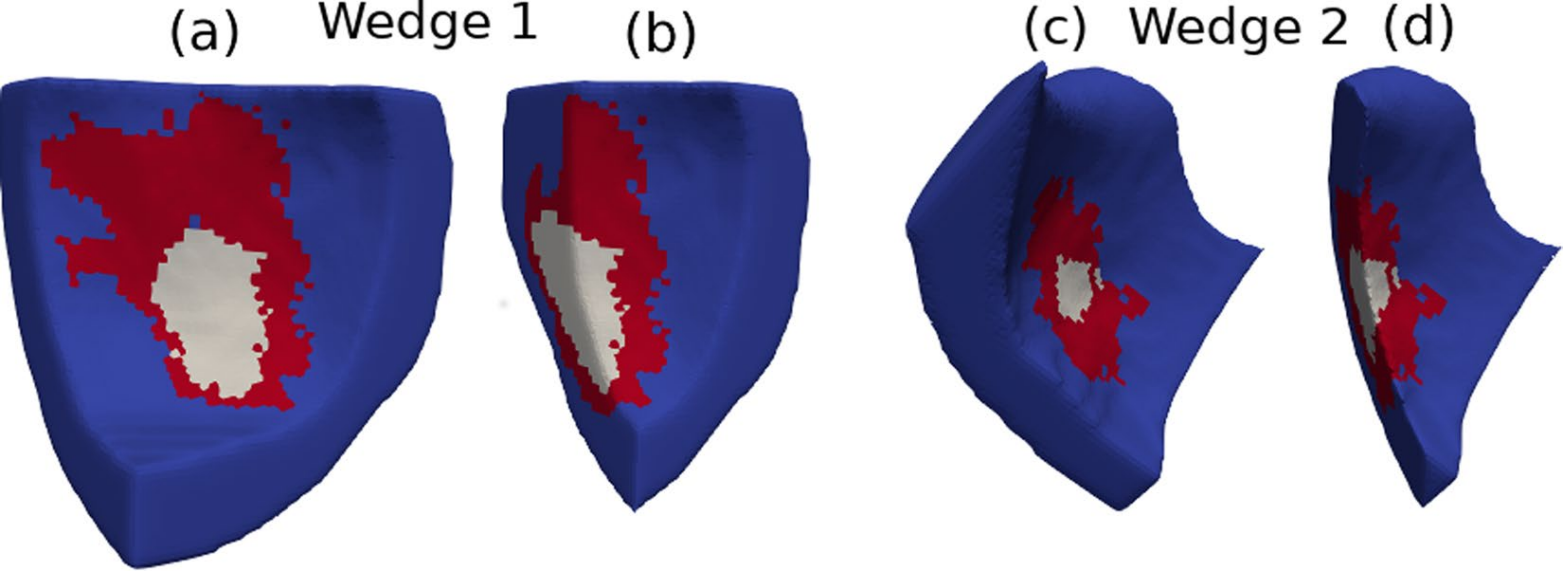
Left ventricular wedges (Wedge 1 and Wedge 2) highlighting the infarct region (in white), peri-infarct zone (in red), and normal tissue (in blue). (**a**) and (**c**) Whole LV Wedges used in the Monte Carlo simulations. (**b**) and **d**) Cuts in the longitudinal direction to facilitate the 3D visualization of the transmural extension of the infarct and peri-infarct zone regions.

In addition, to further reduce the amount of computations in our numerical experiments, we evaluated the probability of each of the two patient-specific infarct regions to become an ectopic pacemaker separately. From the bi-ventricular mesh, we extracted two smaller sub-meshes, i.e., two cardiac wedges, that surrounded each infarct region. The first cardiac wedge is depicted in Fig. 5(a) and contained a large infarct region and PZ. The second wedge is shown in Fig. 5(c) and contained a small infarct scar. Each wedge was built in a way that it contains one of the infarct regions. Its size was chosen to guarantee that healthy tissue surrounds the infarct, i.e., at the borders of the wedge there is healthy tissue and the infarct is at the center of the wedge. In addition, both wedges are transmural cuts, i.e., they include both epi- and endocardial surfaces.

For each wedge, we generated a total of 240 different realizations by varying the fraction *ϕ* of the disconnected volumes (non-conducting material) from 0.64 to 0.69 in steps of 0.01; using two different values of [*ATP*_*i*_] for the myocytes in the center of the scar, 2 and 3 mM; and generating 20 different, non-conducting mazes for each pair (*ϕ*, [*ATP*_*i*_]) of parameters. In the PZ we incorporated a certain degree of fibrosis and ischemia by modulating the parameters [*ATP*_*i*_] and *ϕ* linearly inside the PZ depending on its distance to the center of the scar. Therefore, in the PZ, [*ATP*_*i*_] and *ϕ* linearly changed from their values inside the scar to their values in the healthy surrounding tissue, 6.8 mM and zero, respectively.

It is important to note that because we randomly disconnected volumes to model diffuse fibrosis, each of the 240 realizations of the same geometrical wedge was unique with respect to both the anatomical microstructure and electrophysiological properties. The uniqueness of the microstructure was straightforward and came from the random method of modeling diffuse fibrosis. The uniqueness of the electrophysiological properties came from the interplay of the distinct topology and electrotonic effects between neighboring myocytes^47^. For instance, Fig. 2 presents AP waveforms obtained at different points for the small wedge simulation (SCAR, PZ, and healthy regions) for the pair (*ϕ* =  0, [*ATP*_*i*_] = 3 mM). In the same region, with the same value of [*ATP*_*i*_], different waveforms were found due to the influence of the neighboring myocytes via electrotonic effects. Therefore, the specific topology of fibrosis affected the AP waveforms, conduction velocity and number and locations of the unidirectional blocks of AP propagation in the infarct region due to the different levels of source-sink mismatch^48^.

### Biventricular patient-specific simulations

The Monte Carlo studies performed for each wedge allowed us to statistically verify the genesis of ectopic pacemakers due to micro-reentries in the infarct region. Using these results, we saved two topologies (one for each wedge) that generated micro-reentries and formed ectopic beats. Without changing any characteristics of these models, i.e., keeping the exact topology and electrophysiological properties, we patched each wedge back into the whole biventricular model. The patient-specific heart model was then evaluated using two different initial stimulus protocols, a single point-like stimulus at the apex and multiple point-like stimuli on the endocardial surface, to reproduce the normal excitation via the Purkinje system. It is important to highlight that the use of non-conforming meshes was crucial for this step. This feature of the cardiac simulator^35^ allowed us to patch back the different wedge models in the whole biventricular model without the need for any re-meshing steps.

## Results

In this study, we employed a biventricular model generated from MRI images of a patient with two infarct regions and evaluated whether these regions could interact with an action potential wave to generate ectopic beats. The following sections present the results of the simulations using the wedges presented in Fig. 5 and the whole biventricular mesh obtained from patient MRI images.

### Wedge simulations

We performed Monte Carlo simulations using the two wedge models, as shown in Fig. 5. The pair of parameters (*ϕ*, [*ATP*_*i*_]) was varied to generate different scenarios, as described in the methods section. For the wedge with the large infarct region, 39 realizations of infarctions, out of a total of 240, presented spontaneous pacemaker-like behaviors after a single point stimulus. The mechanism behind the ectopic pacemaker was the appearance of sustained micro-reentries inside the infarct region.

**Figure 6:**
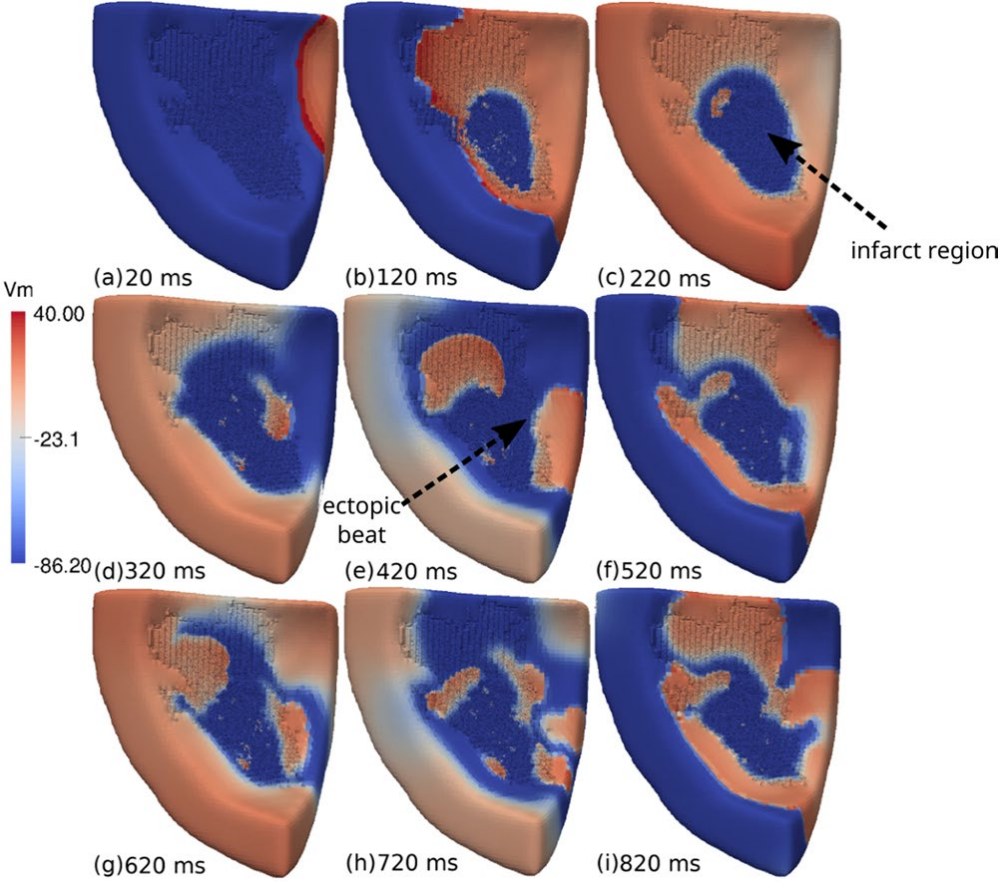
Visualization of the transmembrane potential of one of the simulations performed during Monte Carlo analysis using the ventricular wedge containing a large scar. This is one of the observed sustained reentries with *ϕ* = 0.66 and [*ATP*_*i*_] = 2 mM. (**a**) A single stimulus is applied at the upper-right region of the wedge. (**b**) The AP wave propagates towards the left of the LV wedge. Inside the infarct region we observe some electrical activity due to a fractionated propagation that occurs in the maze formed by non-conducting fibrosis and conducting myocytes. (**c**) The LV wedge is fully depolarized. Micro-reentries lead to the formation of a fractionated spiral wave inside the infarct region. (**d**) The LV wedge begins to repolarize. An AP wave begins to leave the infarct region at its right side. (**e**) An ectopic beat is generated at the right side of the infarct region and propagates towards the healthy tissue. Another ectopic beat leaves the infarct region on the top and enters the peri-infarct zone region. (**f**) The LV wedge begins to depolarize again. Inside the infarct region, micro-reentries persist. (**g**) Another AP wave begins to leave the infarct region at the right side of the infarct region. (**h**) A second ectopic beat is generated at the right side of the infarct region and propagates towards the healthy tissue. (**i**) A complex pattern of AP waves is formed via the interaction between micro-reentries inside the infarct region and AP propagation in the healthy tissue.

A typical pattern of an AP propagation is shown in Fig. 6 (see Supplementary Video 2) for wedge 1 with *ϕ* = 0.66 and [*ATP*_*i*_] = 2 mM at the center of the infarct region. In frame (a), we can observe a single stimulus that was applied at the upper-right region of the wedge. Then, the AP wave propagated towards the left side of the LV wedge. Inside the infarct region, we observed some electrical activity due to the fractionated propagation that occurred in the maze formed by non-conducting and conducting myocytes, as show in frame (b). After 220 ms (c), the LV wedge was fully depolarized, and micro-reentries led to the formation of a fractionated spiral wave inside the infarct region. The LV wedge then began to repolarize, and an AP wave began to leave the infarct region at its right side, as shown in frame (d). After 420 ms (e), an ectopic beat was generated at the right side of the infarct region and propagated towards the healthy tissue. Another ectopic beat left the infarct region from the top and entered the PZ region. The LV wedge began to depolarize again. After 520 ms (f) the micro-reentries persisted inside the infarct region. Another AP wave began to leave the infarct region at the right side of the infarct region, as shown in frame (g). Then, a second ectopic beat was generated at the right side of the infarct region and propagated towards the healthy tissue, as shown in frame (h). A complex pattern of AP waves was formed via the interaction between micro-reentries inside the infarct region and AP propagation in the healthy tissue, as show in frame (i). After approximately 800 ms, the infarct region was able to produce several ectopic beats that propagated throughout the rest of the wedge.

**Figure 7:**
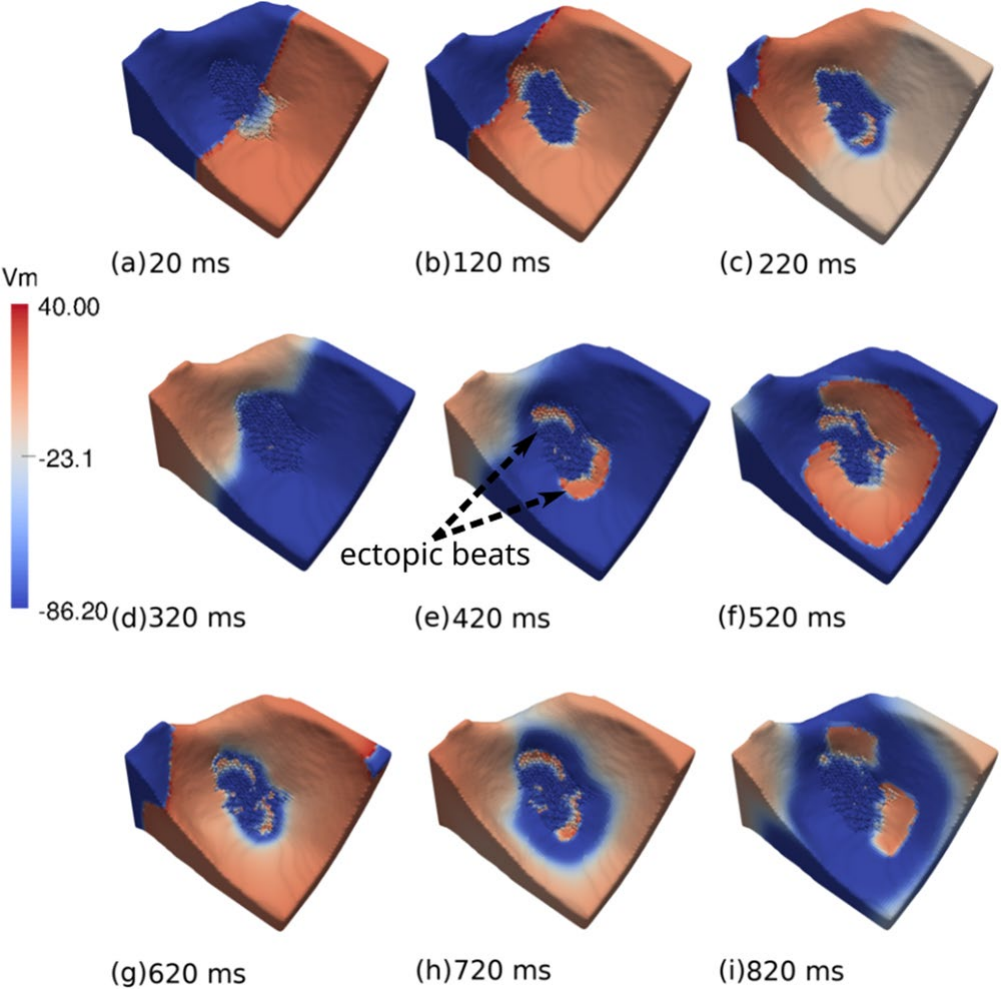
Visualization of the transmembrane potential of one of the simulations performed during Monte Carlo analysis using the ventricular wedge containing the small scar. This is one of the observed sustained reentries with *ϕ* = 0.66 and [*ATP*_*i*_] = 2 mM. (**a**) A single stimulus is applied at the right region of the wedge. (**b**) The AP wave propagates towards the left of the LV wedge. (**c**) The LV wedge is almost fully depolarized. Micro-reentries lead to the formation of a fractionated figure-of-eight pattern inside the infarct region. (**d**) The LV wedge begins to repolarize. (**e**) Two ectopic beats simultaneously leave the infarct region and re-excite the healthy tissue. (**f** and **g**) The LV wedge begins to depolarize again. Inside the infarct region a fractionated figure-of-eight pattern persists (**h**). Healthy tissue repolarizes. (**i**) The sustained fractionated figure-of-eight pattern re-excites the tissue again.

For the wedge containing the small infarct region, only 20 infarct realizations, out of 240, presented spontaneous pacemaker-like behaviors. Nevertheless, as shown in Fig. 7, similar dynamics were observed (see Supplementary Video 3). Once again, the mechanism behind the ectopic activity was the appearance of sustained micro-reentries inside the infarct region.

**Figure 8:**
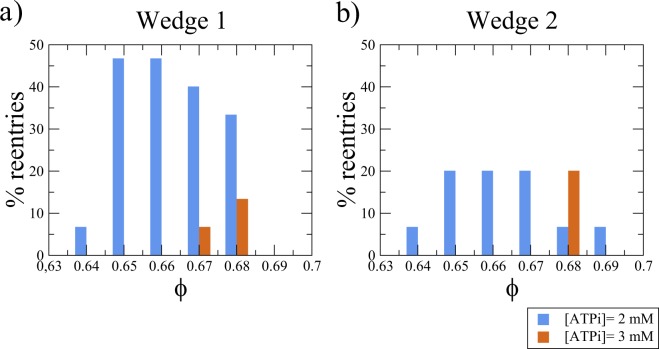
Results obtained from Monte Carlo simulations of the ventricular wedges by varying the percentage of fibrosis, (*ϕ*), and the degree of ischemia in the infarct region ([*ATP*_*i*_]). For each fixed value of *ϕ*, 20 different realizations are generated and simulated, each one with a different topological structure of the fibrotic network. The blue bars show the percentage of sustained reentries observed for different values of *ϕ* with [*ATP*_*i*_] = 2 mM. The orange bars show the number of sustained reentries obtained for different values of *ϕ* with [*ATP*_*i*_] = 3 mM. Reentries are found for values of *ϕ* near the theoretical limit for the percolation threshold, which is equal to 0.68.

Figure 8(a) presents the statistical results of the Monte Carlo study using ventricular wedge 1 with the large scar. The blue bars show the percentage of sustained reentries observed 1 s after a single stimulus with [*ATP*_*i*_] = 2 mM and varying *ϕ* from 0.64 to 0.69 (steps of 0.01). Reentries were found for values of *ϕ* near the theoretical limit for the percolation threshold, which is equal to 0.68^25^. The orange bars show the number of sustained reentries obtained for different values of *ϕ* with [*ATP*_*i*_] = 3 mM. Fewer reentries were found in the last case. The decrease of the percentage of reentries with [*ATP*_*i*_] was associated with the dependence of the APD on the value of [*ATP*_*i*_]. The probability of generating micro-reentries in an infarct region decreased when APD increased, in accordance with our previous observations^25^.

A similar set of simulations was performed using the small wedge (wedge 2), and the results are shown in Fig. 8(b). We observed a similar behavior for the second wedge. Sustained reentries were generated for similar values of *ϕ*, close to the percolation threshold. However, there was a decrease in the total number of wedges with sustained reentries. This reduction in the number of reentries was expected and is mainly related to the size of the infarct. The probability of randomly generating a reentry circuit naturally decreases with the size of the infarct region^27^.

### Biventricular simulations

**Figure 9:**
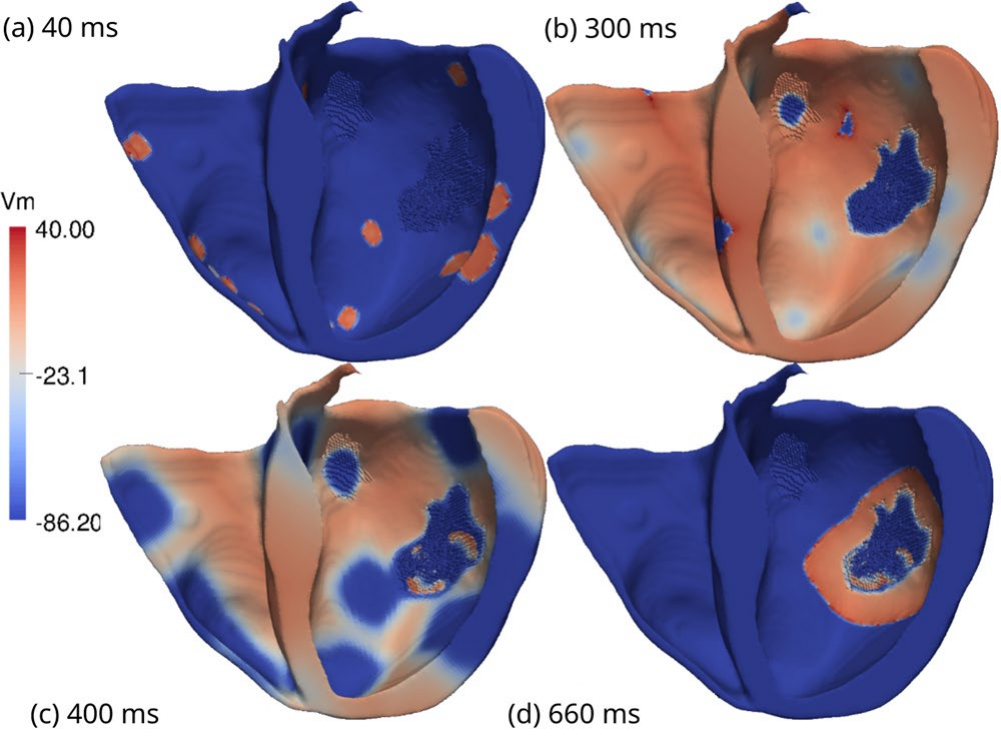
Visualization of the transmembrane potential of one of the simulations performed using the biventricular patient-specific mesh (to facilitate the visualization only half of the ventricles is shown in the figure). The simulation presents sustained reentries with *ϕ* = 0.66 and [*ATP*_*i*_] = 2 mM. (**a**) Multiple stimuli were applied to different subendocardial regions to mimic an activation via the Purkinje network. (**b**) Both ventricles are fully depolarized. No electrical activity inside the small infarct region at the upper region of the LV is found. This behavior holds during the entire simulation. However, inside the larger infarct region we observe persistent electrical activity due to the fractionated propagation of AP waves. (**c**) The ventricles begin to repolarize. Inside the large infarct region micro-reentries lead to the formation of two fractionated spiral waves. (**d**) An ectopic beat is generated at the right side of the large infarct region and propagates towards the healthy tissue.

After the set of simulations using cardiac wedges shown in the previous section were carried out, we performed simulations using the full biventricular mesh. We chose specific configurations of the fibrotic structure that generated an ectopic beat in the previous wedge simulations and incorporated these infarct patterns back into the full biventricular mesh. Both wedge models used for the full biventricular simulations were generated using *ϕ* = 0.66 and [*ATP*_*i*_] = 2 mM. The patient-specific heart model was then evaluated using two different initial stimulus protocols; a single point-like stimulus at the apex (see Supplementary Video 4) and multiple point-like stimuli on the endocardial surface, to reproduce the normal excitation via the Purkinje system (see Fig. 9 and Supplementary Video 5). For these two simulations, the results were similar. Sustained reentries were found in the large infarct region. These micro-reentries were the mechanism for the genesis of ectopic beats that came out of the large infarct region (see Fig. 9), spread through the whole heart and induced a cardiac arrhythmia (see Supplementary Videos 3 and 4).

A different trend was observed for the small infarct because it only behaved like an ectopic pacemaker with a single point-like stimulus at the apex (see Supplementary Video 3). For simulations with multiple point-like stimuli on the endocardial surface, micro-reentries were not observed in the region of the small infarct, despite the fact that we did not change any of the characteristics of this model, i.e., we kept the exact topology and electrophysiology properties as those that gave rise to micro-reentries in the wedge simulations. This is a clear suggestion that the path and direction by which the AP wave arrives at the infarct region may also play important roles in the genesis of micro-reentries and ectopic beats.

On the other hand, the topology chosen for the large infarct region was very robust in terms of pro-arrhythmic features and did not show a dependency on the stimulation protocol. Figure 9 shows the transmembrane potential of one simulation performed using the whole biventricular, patient-specific mesh. In frame (a), we observe the application of multiple stimuli on different subendocardial regions. The main idea behind this protocol is to mimic an activation via the Purkinje network^43,49^. After 120 ms, both ventricles were fully depolarized, which is in close agreement with the patient QRS duration of 100 ms, as recorded by an ECG. During this period, there was no electrical activity inside the small infarct region at the upper region of the LV. This behavior held during the entire simulation. However, inside the large infarct region, we observed some electrical activity due to the fractionated propagation of AP waves. Frame (c) shows that after 400 ms, the ventricles began to repolarize. Inside the large infarct region, micro-reentries led to the formation of two fractionated spiral waves, in a figure-of-eight pattern. After 660 ms, an ectopic beat was generated at the right side of the large infarct region and propagated towards the healthy tissue.

## Discussion

### The trigger

Computational modeling has been used before to study the relationship of arrhythmias and fibrosis or other non-conducting media in cardiac tissue^16–23,28,31^. However, only a few studies have focused on the genesis of ectopic beats or premature ventricular contractions (PVCs)^25,26,30^. We quote Boineau and Cox^13^ to highlight how our work differs from other important studies: “Our studies differ from these in that premature beats were not used to evoke re-entry. We were primarily concerned with the spontaneous development of PVCs as a natural consequence of the single intervention of coronary occlusion”. Therefore, we did not use S1-S2 or fast pacing stimulation protocols^50^. After a single stimulus, we observed how the AP wave fractionated inside the infarct region, gave rise to micro-reentries, and re-excited the heart via ectopic beats that left the PZ of the infarct region.

It is worth mentioning that the main goal of the simulations performed in this work was to verify whether the mechanism of micro-reentries, proposed half a century ago, could generate ectopic beats and trigger arrhythmias using patient-specific data of myocardial infarction. However, this is a purely theoretical work, and many simplifications and hypotheses were adopted in the development of the models. One important hypothesis is that the scar and PZ regions detected by LE-MRI were used as markers for severe and moderate ischemic regions, respectively. Therefore, our results reflect cases of recurrent MI, where fibrosis and ischemic myocytes are found next to each other. However, other interpretations are possible. Fibrosis, as detected by LE-MRI, indicates the locations of necrotic cells at the time of the acute phase of the disease. Therefore, we can also interpret our results as simulations of the acute MI episode of the patient, where necrosis and ischemic myocytes are found next to each other. In summary, we can use our simulations to look ahead and predict the scenarios that can be pro-arrhythmic in situations of recurrent MI or look into the past, and study how dangerous untreated acute MI can be.

Different mechanisms have been proposed to explain the appearance of ectopic beats near infarct regions, such as abnormal automaticity, triggered activity, micro-reentries, or even the combination of some of these three. For instance, experimental and computational studies have suggested that post-acidosis arrhythmias during acute reperfusion may be triggered by delayed after-depolarizations (DADs)^51,52^. Computer simulations have also shown that DADs can overcome the source-sink mismatch that arises from inter-cellular coupling and produce a PVC at the tissue level either by increasing the source via synchronization^53^ or by decreasing the sink via fibrosis^54^. Therefore, in the near future we plan to improve our model, that here focus only on the micro-reentry mechanism, to be able to study the combination of the different aforementioned mechanisms in the triggering of PVCs.

### Biological uncertainties and complexity

In terms of the methods used in this work, the Monte Carlo approach was crucial to evaluate the uncertainties of our patient-specific model. In particular, those associated with the intricate topology and heterogeneity of the electrophysiology properties of the infarct and PZ regions. This method allowed the presentation of results in a more solid and statistical framework. Instead of a binary result for a certain pair of values (*ϕ*, [*ATP*_*i*_] ) we found, as a result, the probability of an infarct to behave as an ectopic pacemaker. It is worth noting that the strategy to perform Monte Carlo simulations using first tissue wedges containing scars proved to be successful. With this scheme, we narrowed down the number of simulations using the full biventricular mesh; we were able to generate ectopic beats on the first try using two different stimulation protocols (apex stimulation and Purkinje-like endocardial stimulation). It is important to mention that the use of non-conforming finite volume meshes was crucial for this strategy. This feature of the cardiac simulator^35^ allowed us to extract cardiac wedges and later patch them back into the whole biventricular model without the need for any re-meshing steps.

### Reentry patterns

It is interesting to note that in recent animal experiments most of the reentry activity measured near infarct regions was similar to a figure-of-eight pattern^55^. Likewise, our simulations, see Fig. 7(h,i), also presented sustained micro-reentries with a similar figure-of-eight pattern. Nevertheless, quite complex and chaotic patterns were also present, see, for instance, Fig. 6(h). In addition, our final biventricular simulation showed that the ectopic beat could also be very similar to a focus origin or point-like source, a pattern that could be easily misinterpreted as coming from an abnormal automaticity, see Fig. 9(d). This shows how challenging it is to demonstrate the micro-reentry mechanism in animal experiments or clinical studies.

### Simple model but complex relations

In this work, we used simple computational models to reproduce ischemia and diffuse fibrosis. Nevertheless, the results of this combination are far from trivial. We observed that ectopic beats were generated for a range of values of the percentage of fibrosis, which was near a topological metric known as the percolation threshold, confirming previously reported results^25–27^. Figure 8 shows that the size of the infarct is also an important parameter for the appearance of reentries, which is in agreement with previous studies^56^. From these results, we observed that the probability of generating ectopic beats is higher in cases of severe ischemia, with [*ATP*_*i*_]  values in a range that is likely to occur during acute ischemia^40,41^. In addition, although our results suggest that the two investigated parameters, the percentage of fibrosis *ϕ* and degree of ischemia, are relevant in the genesis of ectopic beats near the infarct region, the direction of the approaching AP wave also played an important role. This dependence was observed in cases of small infarct regions. By changing only the location of the initial stimulations, the same infarct model (with the same topology and electrophysiology) that gave rise to micro-reentries in the wedge simulations was electrically silent in the biventricular simulation with multiple point-like stimuli on the endocardial surface. This dependence on the direction of the approaching AP wave is in accordance with our previous results^26,41^.

### A new protocol, its limitations and future works

The use of the aforementioned models and methods allowed the first assessment of a protocol based on cardiac imaging and computational modeling techniques that has the potential to test and quantify the pro-arrhythmic nature of some specific regions of the heart. The protocol has, as inputs: 1) data of late-enhancement MRI from a patient who experienced a myocardial infarction; 2) modern models for the electrophysiology of cardiac myocytes under normal and ischemic conditions^38,40^; and 3) a simple model of diffuse fibrosis. Each one of these can be further improved or complemented in future works. To reproduce the shortening of wavelengths due to ischemia, we focused on hypoxia to reduce the number of parameters to be investigated by our Monte Carlo analysis. In the near future, we plan to study how hyperkalemia and acidosis would affect our results. We used a very simple model of diffuse fibrosis. Different models could be considered to describe compact, interstitial or patchy fibrosis or to include myocyte-fibroblast connections^22,31,45^. Nevertheless, it is worth mentioning that we obtained similar results, in terms of micro-reentry and the generation of ectopic beats, in a previous work that used a more detailed and realistic model of diffuse fibrosis by considering a fine sub-cellular spatial resolution of 8 *μm*^26^. In addition, it would be interesting to study how cellular uncoupling due to gap-junction remodeling in ischemic regions^57^ could influence the micro-reentry mechanism. Finally, the coupling of the Purkinje network to the ventricles was simulated by multiple point-like stimuli. In the near future, we will couple our 3D biventricular model to a Purkinje network model^58^.

### A promising tool for the planning of clinical interventions

Our results align with recent findings of clinical electrophysiology studies that indicate the ablation procedure for the treatment of ventricular tachycardia (VT) or fibrillation (VF). For instance, the clinical study presented by Peichl *et al*.^59^ concludes that catheter ablation of ectopic beats triggering VT or VF could successfully abolish these arrhythmias. Different types of techniques of ablation for VT exist. The multicenter study presented in Di Biase *et al*.^60^ compared mapped VT (induction of VT via fast pacing) and the substrate or homogenization technique, which maps the electrical activity during sinus rhythms. Mapped VT is used to localize macro-reentries, usually believed to be associated with an isthmus, i.e., a conducting path that connects two opposite sites of a non-conducting scar. After the isthmus is localized, this conductive pathway is disrupted by, for instance, a linear ablation that transversely crosses it. The substrate technique identifies all regions with fractionated and low-amplitude electrograms inside both scar and PZ regions. These are the targets for ablation. The results reported in Di Biase *et al*.^60^ suggested that the substrate technique was superior to the mapped VT. We can interpret this result as additional clinical evidence that supports the micro-reentry mechanism. In fact, using our diffuse fibrosis model and, replacing every active cell with fibrosis (via ablation), we would completely suppress any electrical activity inside the infarct region; whereas, by splitting the infarct region in half (via a linear ablation), we would only reduce the probability of generating micro-reentries. Nevertheless, the generation of new fibrotic regions via ablation can also be pro-arrhythmic. Fibrosis is a dynamic process, and recent reviews on this topic highlight the disappointing efficacy of VT ablation in structural heart disease and note that new technologies are needed to optimize and improve this procedure^61,62^. In this direction, the protocol and methods described in this work, which combine imaging techniques and computational models, could be extremely useful for the planning and optimization of clinical interventions, such as catheter ablation. It is important to note that the pipeline of methods proposed in this work can be promptly used for the study of other patients with a recent history of myocardial infarction and available cardiac MRI data. Therefore, a future study that involves more patients might prove the potential clinical prognostic value of the protocol proposed in this work which combines patient-specific data, uncertainty evaluation and computational simulations.

## Conclusion

In this work we investigated, using a patient-specific heart model, the mechanism of micro-reentry in the generation of ectopic beats near infarct areas. This mechanism was described and proposed half a century ago as a result of clinical and animal studies^13–15^. However, the lack of techniques that provide the needed spatial resolution in animal experiments or clinical studies has prevented a final proof of this mechanism^63^. We believe the results presented in this work are an important step toward this goal. Our study uses a heart model based on patient MRI data that contains detailed 3D information of the human ventricle anatomy and the shape and location of the myocardial infarction as well as an established electrophysiology model of the human ventricle that is modified to describe different levels of ischemia. The generated model was able to reproduce the genesis of an ectopic pacemaker near an infarct region. After normal activation via multiple sites on the endocardial surface, which mimics activation via the Purkinje network, the AP propagation fractionates inside the infarct region and gives rise to sustained micro-reentries. In turn, this sustained activity leaves, periodically, the infarct region and re-excites the surrounding healthy tissue, i.e., micro-reentries transform the infarct region into an ectopic pacemaker. Finally, we believe that the validation process of the micro-reentry mechanism will benefit from the combination of data from late-enhancement MRI and the computational models used in this work with data from new electrical mapping techniques^55,64^.

## Electronic supplementary material

Supplementary Video 1

Supplementary Video 2

Supplementary Video 3

Supplementary Video 4

Supplementary Video 5

## Data Availability

All data generated or analyzed during this study are included in this published article (and its supplementary information files).
